# The importance of scale-dependent ravine characteristics on breeding-site selection by the Burrowing Parrot, *Cyanoliseus patagonus*

**DOI:** 10.7717/peerj.3182

**Published:** 2017-04-26

**Authors:** Myriam Ramirez-Herranz, Rodrigo S. Rios, Renzo Vargas-Rodriguez, Jose-Enrique Novoa-Jerez, Francisco A. Squeo

**Affiliations:** 1 Departamento de Biología, Universidad de La Serena, La Serena, Chile; 2 Instituto de Ecología y Biodiversidad, Casilla 599, La Serena, Chile; 3 Programa de Investigación Ecológica en Zonas Áridas (PIEZA), Universidad de la Serena, La Serena, Chile; 4 Departamento de Ciencias Sociales, Universidad de La Serena, La Serena, Chile; 5 Centro de Estudios en Zonas Áridas (CEAZA), La Serena, Chile; 6 Current affiliation: Universidad San Francisco Xavier de Chuquisaca, Bolivia

**Keywords:** Nesting site, Burrowing bird, Intrinsic morphological characteristic, Foraging, Landscape variables

## Abstract

In birds, the environmental variables and intrinsic characteristics of the nest have important fitness consequences through its influence on the selection of nesting sites. However, the extent to which these variables interact with variables that operate at the landscape scale, and whether there is a hierarchy among the different scales that influences nest-site selection, is unknown. This interaction could be crucial in burrowing birds, which depend heavily on the availability of suitable nesting locations. One representative of this group is the burrowing parrot, *Cyanoliseus patagonus* that breeds on specific ravines and forms large breeding colonies. At a particular site, breeding aggregations require the concentration of adequate environmental elements for cavity nesting, which are provided by within ravine characteristics. Therefore, intrinsic ravine characteristics should be more important in determining nest site selection compared to landscape level characteristics. Here, we assess this hypothesis by comparing the importance of ravine characteristics operating at different scales on nest-site selection and their interrelation with reproductive success. We quantified 12 characteristics of 105 ravines in their reproductive habitat. For each ravine we quantified morphological variables, distance to resources and disturbance as well as nest number and egg production in order to compare selected and non-selected ravines and determine the interrelationship among variables in explaining ravine differences. In addition, the number of nests and egg production for each reproductive ravine was related to ravine characteristics to assess their relation to reproductive success. We found significant differences between non-reproductive and reproductive ravines in both intrinsic and extrinsic characteristics. The multidimensional environmental gradient of variation between ravines, however, shows that differences are mainly related to intrinsic morphological characteristics followed by extrinsic variables associated to human disturbance. Likewise, within reproductive ravines, intrinsic characteristics are more strongly related to the number of nests. The probability of producing eggs, however, was related only to distance to roads and human settlements. Patterns suggest that *C. patagonus* mainly selects nesting sites based on intrinsic morphological characteristics of ravines. Scale differences in the importance of ravine characteristics could be a consequence of the particular orography of the breeding habitat. The arrangement of resources is associated to the location of the gullies rather than to individual ravines, determining the spatial availability and disposition of resources and disturbances. Thus, nest selection is influenced by intrinsic characteristics that maximize the fitness of individuals. Scaling in nest-selection is discussed under an optimality approach that partitions patch selection based on foraging theory.

## Introduction

Nest-site selection in birds is a key life history event that directly affects their reproductive success (Walsberg, 1981; Li & Martin, 1991; White Jr, Brown & Collazo, 2006). Several factors affect nest-site selection including microclimatic variables around the nest (e.g., temperature, humidity, solar radiation) as well as intrinsic variables (e.g., orientation, cavity size and substrate composition). Intrinsic factors and ambient temperature determine nest microclimate and consequently, can alter cost of reproduction and chick development (Webb, 1987; Wiebe, 2001; Rhodes, O’Donell & Jamieson, 2009; Ardia, Perez & Clotfelter, 2006; Yuan et al., 2006; Hilde et al., 2016). Accordingly, birds choose nest orientation in relation to sun exposure to optimize reproductive success concurrent to their abiotic environment, especially in extreme areas such as deserts (Ardia, Perez & Clotfelter, 2006; Salaberria et al., 2014; Matthew, 2013). Nest-site selection is also influenced by those biotic characteristics that alter reproductive potential, such as food availability, susceptibly to predation and anthropic disturbances (Haynes et al., 2014; Nilsson, 1984; Bruggeman et al., 2015). Human disturbances at nesting-habitats act as additional factors that can generate loss of potential nest-sites with major impact at the population level (Hill et al., 1997; Lowe, Rogers & Durrant, 2014). The level of impact will depend on the birds’ degree of specialization to their nesting resources (Martin, Aitken & Wiebe, 2004). For example, nest-specialists such as cavity-nesting species are constrained by the availability of cavities or adequate substrate for nesting (Heneberg, 2003; Heneberg, 2013b). Thus, they are greatly affected by human induced changes when they reduce adequate nesting-habitat availability.

Cavity-nesting birds can be divided into primary cavity-excavators, which build their own cavities in specific substrates, such as burrowing birds that excavate their nest in ravines with a specific soil composition; and secondary cavity-nesters, those that use pre-existing cavities usually built by other species (Eberhard, 2002; Cockle, Martin & Weibe, 2011). Cavity nesters usually select substrate or cavity characteristics that give the nest adequate microclimatic conditions and low predation risk (White, Bartholomew & Kinney, 1978; Wickler & Marsh, 1981; Hooge, Stanback & Koenig, 1999; Brightsmith, 2005; Salaberria et al., 2014; Bruggeman et al., 2015). Cavity-characteristics, such as greater verticality and height above ground, reduced entry size or increased cavity depth, can limit predator access (Webb, 1987; Li & Martin, 1991; Newton, 1994; Hooge, Stanback & Koenig, 1999; Jenkins, 2000; Heneberg, 2013a). Commonly, nest characteristics associated to a reduction in predation risk also confer a highly stable microclimate, which generally increases reproductive success (Rhodes, O’Donell & Jamieson, 2009). Furthermore, burrowing birds depend heavily on the availability of a suitable nesting substrate. Those species choose optimal substrate according to their composition, soil particle size, packing density and permeability; features that play an important role in incubation and brooding, which often limit their distribution and/or breeding densities (Eberhard, 2002; Martin, Aitken & Wiebe, 2004; Cornelius, 2008; Salinas-Melgoza, Salinas-Melgoza & Renton, 2009; Heneberg, 2013a).

Because a primary cavity excavator strategy allows individuals to build nests near conspecifics without relying on previously existing nesting resources, Eberhard (2002) postulates that this strategy is more likely to be related to the ability to form breeding colonies compared to a secondary cavity-nester strategy. Coloniality consists of high density nesting within a restricted resource-based territory for breeding that improves detection of and defense against predators, promotes group feeding and enhances conspecific knowledge of favorable habitat for breeding (Wagner & Danchin, 2003; Eberhard, 2002; Sachs et al., 2007). Therefore, this breeding aggregation requires the concentration of adequate environmental elements for cavity nesting at a specific area (Masello & Quillfeldt, 2002). Factors that promote conspecific nest proximity and large colonial breeding have been scarcely studied in gregarious cavity-excavators. Most of our knowledge of cavity nest-site selection has emerged from studies based on secondary cavity-nesters focused on resource competition, or on their dependence on excavator species (Ardia, Perez & Clotfelter, 2006; Cornelius et al., 2008; Rhodes, O’Donell & Jamieson, 2009; Salinas-Melgoza, Salinas-Melgoza & Renton, 2009; Cockle, Martin & Weibe, 2011; Salaberria et al., 2014). Moreover, studies of primary cavity excavators have centered only of solitary forest-dwelling species, such as North American and European woodpeckers, or European burrowing birds; such as colonial European bee-eaters. These last studies, focused on soil composition and human disturbance, all of which have been analyzed separately (Hooge, Stanback & Koenig, 1999; Heneberg, 2009; Kosiński et al., 2011; Heneberg, 2013a; Smalley et al., 2013a; Tella et al., 2014; Hoi, Kristofik & Darolov, 2015; Lorenz et al., 2015). Analogous studies with Neotropical birds are lacking.

Among birds, Psittacidae is the taxa with the greatest number of cavity-nesting species; most are obligate secondary cavity-nesters and just a few species are excavators (Brightsmith, 2005; Juniper & Parr, 2010). One of those few primary cavity excavator parrots is *Cyanoliseus patagonus* (The Burrowing Parrot), which has the unique ability to nest on fluvial ravines forming the largest breeding colonies ever recorded for parrots (Masello & Quillfeldt, 2012). Their gregarious breeding strategy could increase selection pressures by conspecifics for choosing an adequate ravine for entire colonies (Eberhard, 2002). This species generally requires sandstone, limestone or dirt-soil cliffs to excavate their nest-burrows (Masello et al., 2006). Therefore, using this peculiar species we assess the importance of extrinsic and intrinsic ravine characteristics on nest-site selection. Specifically, we test the hypothesis that within ravine characteristics are more important in determining nest site selection compared to landscape level characteristics. We predict that differences between selected and non-selected breeding ravines should emerge by the disparity in orientation, substrate type and ravine size rather than by differences in the available resources found in the proximate environment where ravines are immersed (e.g., proximity to food and water). As a consequence, differences should be related to reproductive success. In addition, we examined if the proximity to human influence affects selection of reproductive ravines, in which case ravines selected for reproduction should be located further away from urban areas and roads compared to non-reproductive ravines.

## Materials & Methods

### Study species

*Cyanoliseus patagonus bloxami*, Olson, 1995, is one of the four subspecies of Burrowing Parrots and is the only representative of the species in Chile. The Burrowing Parrot has suffered a clear reduction in its range since the early nineteenth century, mainly due to loss and degradation of its natural habitat, leading to the fragmentation of their populations (Galaz, 2005). Currently, *C. patagonus bloxami* is the most endangered subspecies and is divided into two isolated populations located north and south within central Chile. In Chile, the subspecies is classified as endangered to the populations of the north, and those of the south are classified as threatened. The main causes of its decline are habitat loss and poaching (Galaz, 2005; Vargas-Rodríguez & Squeo, 2014). The northernmost population inhabits a semi-desert ecosystem, while the southern population inhabits a Mediterranean habitat (Masello & Quillfeldt, 2012; Vargas-Rodríguez & Squeo, 2014). The northern population is known for living under extreme environmental conditions, where resource availability is scarce. Studies with other subspecies from Argentina have shown that resource availability (water and food) at nesting ravines influences population sizes of this species (Masello & Quillfeldt, 2012). The species has a very extensive home range, making long daily trips to different sites such as foraging areas, reproductive ravines, water bodies and roosting areas. For example, individuals can make daily trips from ravines to their feeding areas, moving an average distance of 40–60 km. Colonies are located close to water bodies and roosting areas, in which at sunset breeding colonies cluster to rest, are located away from reproductive ravines (Masello et al., 2006; Vargas-Rodríguez & Squeo, 2014). The burrowing parrots have a monogamous breeding system with intensive biparental care and high coloniality (Juniper & Parr, 2010). At a ravine, individual pairs excavate nests used in previous seasons enlarging them every year. Nests have an average depth of 1.5 m ending in an incubation chamber where eggs are laid on bare ground. Each nest is occupied by a single pair that lays between two to five eggs per season. The species is very sensitive to disturbance during the incubation period (Masello & Quillfeldt, 2004; Masello & Quillfeldt, 2012).

**Figure 1:**
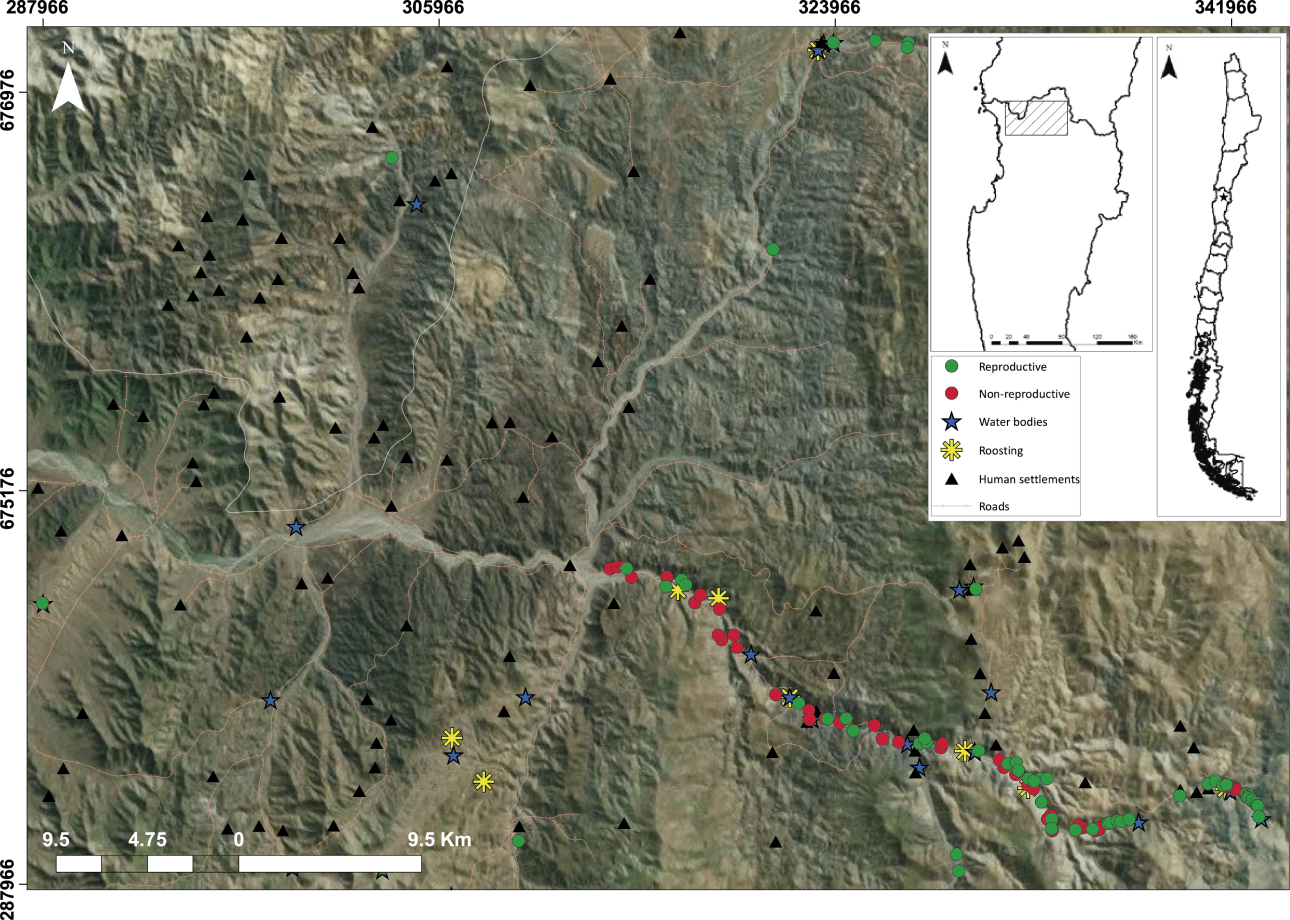
Study area (*ca*., 1,300 km^2^) in which landscape elements are using by the species like reproductive ravines (green circles), non-reproductive ravines (red circles), water bodies (blue stars) and roosting (yellow asterisks); And anthropic elements, roads (orange lines) and human settlements (yellow triangles).

### Study area

We conducted the study during the breeding seasons of 2013 and 2014 in northern Chile where the northernmost population of *C. patagonus bloxami* is located. This region is composed of an arid ecosystem dominated by xerophytic vegetation where fluvial ravines are positioned on the slopes located on both sides of creeks. We covered an area of *ca.* 1,300 km^2^, within the first 54 km along the Andean foothills, covering the entire altitudinal range of the species (180–2,000 m) (Fig. 1). Mean annual precipitation is lower than 80 mm, and heavily influenced by ENSO events (Squeo et al., 2009).

Ravines can be classified as reproductive and non-reproductive depending on the presence of nests. In turn, reproductive ravines can be active or inactive, depending on the presence of individuals along a given breeding season. We quantified 12 variables in all ravines present in the study area (*n* = 105), which were accurately georeferenced (UTM WGS84). Seven of these variables were related to morphological and intrinsic characteristics of ravines: area, height, slope, orientation, type of substratum, geological origin and the nature of the ravine (origin geological or artificial of the ravine). Every ravine was photographed with its respective reference scale in order to calculate its area and maximum height (represented from the base of ravine to the highest point) using Photoshop. A compass was used to determine orientation and a clinometer to record the slope at the midpoint of every ravine. In order to evaluate the effect of the type of ravine, we determined the substrate based on its geomorphological and morphodynamic origin, in addition to the presence and size of rocks (Fairbridge, 1968; Huggett, 2003). Moreover, ravines were categorized depending on whether they were of natural origin (geological processes) or artificial origin (extraction of gravel by human activity).

Three of the variables analyzed were related to the presence and availability of resources in the study area. We estimated the distance to water bodies and roosting areas (protected valley bottoms with high densities of tall cactus where individuals perch) closest to each ravine using Google Earth as a proxy of the availability of these resources. Food availability was estimated based on the abundance of plant species that are the main food source of *C. patagonus bloxami* (e.g., *Balsamocarpon brevifolium*, *Cordia decandra* and *Krameria cystoidea*) (Vargas-Rodríguez & Squeo, 2014) in a radius of 50 m around each ravine. Abundance data of each plant species were summed to form a single value of food in the areas near the ravine. In addition, we calculated the distance from ravines to the nearest places with human disturbance (e.g., human settlements: settlements that house between one and five basic family houses of rural type that are dedicated to cattle raising; and roads: unpaved rural roads transited with medium and low frequency of vehicles) in order to evaluate the effect of landscape modification on nest-site selection. Finally, to evaluate the relationship between nest-site selection and fitness, we measured the number of nests and egg production (with or without eggs) for each reproductive ravine during both reproductive seasons.

### Statistical analyses

To determine differences between non-reproductive and reproductive ravines and between active and inactive ravines in intrinsic and extrinsic variables, independent PERMANOVAs (Anderson & Walsh, 2013) were performed for both sets of ravines using 1,000 permutations for each comparison. We used all continuous variables in the analyses because none had a high degree of correlation (<*r*_*s*_ = 0.9) as indicated by independent spearman correlation tests. Subsequently, to graphically observe the interrelationship among variables that explain the difference between groups of ravines we performed non-metric multidimensional scaling (NMDS) using 9,999 permutations, based on a Euclidean distance matrix to preserve the ordering relationships among objects in the specified number of axes. This matrix results in a less deformed representation of the distance relationships among objects than other distance methods in the same number of dimensions (Borcard, Gillet & Legendre, 2011). To assess whether there was a significant relationship among the ordination axis scores and ravine characteristics and the percent of variance explained by each variable (*R*^2^), we used the *envfit()*-function with 9,999 permutations from the vegan package. Moreover, to determine if characteristics of active ravines are related to the number of nests, a generalized linear model (GLM) was performed using a Poisson distribution and a log-link function. The model included seven continuous variables that were relevant to the number of nests on a ravine (area, height, distance to water bodies, food availability, distance to roosts, distance to human settlements and distance to roads). In order to test for variable significance we conducted Likelihood Ratio Tests (LRT). In order to test if characteristics of active ravines are related to the probability of laying eggs at a ravine, a similar analysis, to the one mentioned above was conducted, except that the GLM model was performed using a Binomial distribution and a logit-link function with the variable with or without eggs as the response. All statistical analyses mentioned previously were performed using the R statistical environment ver. 2.15.3 (R Core Team, 2013). Finally, mean orientation angle was calculated as a measure of ravine orientation using circular statistics and homogeneity distribution based on a Rayleigh test (Zar, 1984). This analysis was performed using the software Oriana ver. 4. (Kovach, 2014).

## Results

### Reproductive and non-reproductive ravine comparison

**Figure 2:**
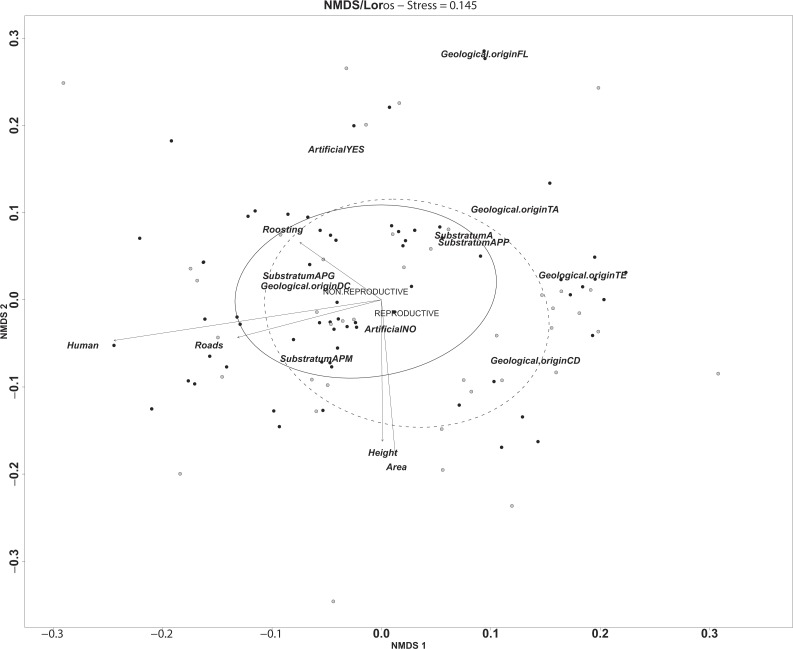
Representation of the multidimensional environmental gradient of variation between non-reproductive ravines (black circles) and reproductive ravines (white circles) where the axes represent the vectors of significant variables in environmental space. Ellipses determine the distribution of the each ravine in the gradient (non-reproductive ravines solid and reproductive ravines dashed lines). Continuous variables are represented by vectors that determine the distance and direction of each variable in the environmental gradient. Categories for geological origin are shown in the environmental gradient without vector representation (APG = sand with big stones; APM, sand with medium stones; APP, sand with small stones; A, sand without stones; CD, cones of dejection, DC, colluvial deposits, FL, flow sandy slope, TA, alluvial terrace; TE, talud erosion).

There are significant differences between non-reproductive and reproductive ravines in their intrinsic and extrinsic characteristics (*F* = 3.5, *df* = 1, *p* = 0.0049); however, we found no differences between reproductively active and reproductively inactive ravines (*F* = 1.35, *df* = 1, *p* = 0.26). Of the eleven variables considered in this analysis nine explained significant variance across the non-reproductive and reproductive ravine gradient (Table 1). The variables that explain variation are related to intrinsic morphological characteristics such as, area, substrate or geological origin and extrinsic variables associated to human disturbance, like distance to human settlements had the greatest explanatory power (Table 1). On average, reproductive ravines are larger, taller and have higher slope than non-reproductive ravines. In addition, they are closer to human settlements and roads (Table S1, Fig. 2).

**Table 1:** Results for the fit between the ordination gradient across non-reproductive and reproductive ravines generated using a NMDS and each of the ravine characteristics considered in the study. The goodness of fit statistic is represented by the squared correlation coefficient (*R*^2^), which represents the percent of variance explained by a variable. Significance of fitted variables is based on 9,999 permutations of these variables and is highlighted by an asterisk. Type of variable refers to elements associated to the intrinsic characteristics of ravine or elements at the landscape level.

Variable	Type of variable	*R^2^*	*p*-value
Area	Intrinsic	0.22	0.001*
Height	Intrinsic	0.19	0.002*
Slope	Intrinsic	0.032	0.182
Nature of ravines	Intrinsic	0.22	0.001*
Geological origin	Intrinsic	0.46	0.001*
Substrate	Intrinsic	0.34	0.001*
Distance to water bodies	Extrinsic	0.024	0.309
Food availability	Extrinsic	0.06	0.045*
Distance to roosts	Extrinsic	0.07	0.029*
Distance to roads	Extrinsic	0.13	0.002*
Dist. to human settlements	Extrinsic	0.44	0.001*

The multidimensional environmental gradient of variation between ravines revealed by the NMDS shows that the height and area of the ravine and distance to roads and human settlements (urbanization) are the variables that generate the gradient across sites. Moreover, distance to human settlements explained most of the variation among ravines (Fig. 2). Although reproductive ravines tend to be closer to populated areas, differences with non-reproductive ravines are more in line with ravine size (height and the area) indicating that reproductive ravines are larger than non-reproductive ravines. The type of geological origin is highly dispersed throughout the environmental gradient, so its importance in differentiation between ravines is variable. Specific categories, however, are more present in reproductive that non-reproductive ravines. For example, cones of dejection (CD) are more common in reproductive ravines. These cones are alluvial fans with very steep slopes that are generally higher and narrower than a fan, and are composed of coarser and thicker material believed to have been deposited by large streams (Herrmann & Bucksch, 2013). Moreover, all sites in study area with alluvial terrace are active reproductive ravines (Fig. S1). Importantly, the ravines of anthropogenic origin (sand and gravel extraction) present in the area are used more frequently significantly (*R*^2^ = 0.22, *p* = 0.001). The use of this type of ravines for reproduction reveals the adjustability of this species to human pressure. Food availability near reproductive ravines and distance to water bodies were less important for ravine selection.

**Figure 3:**
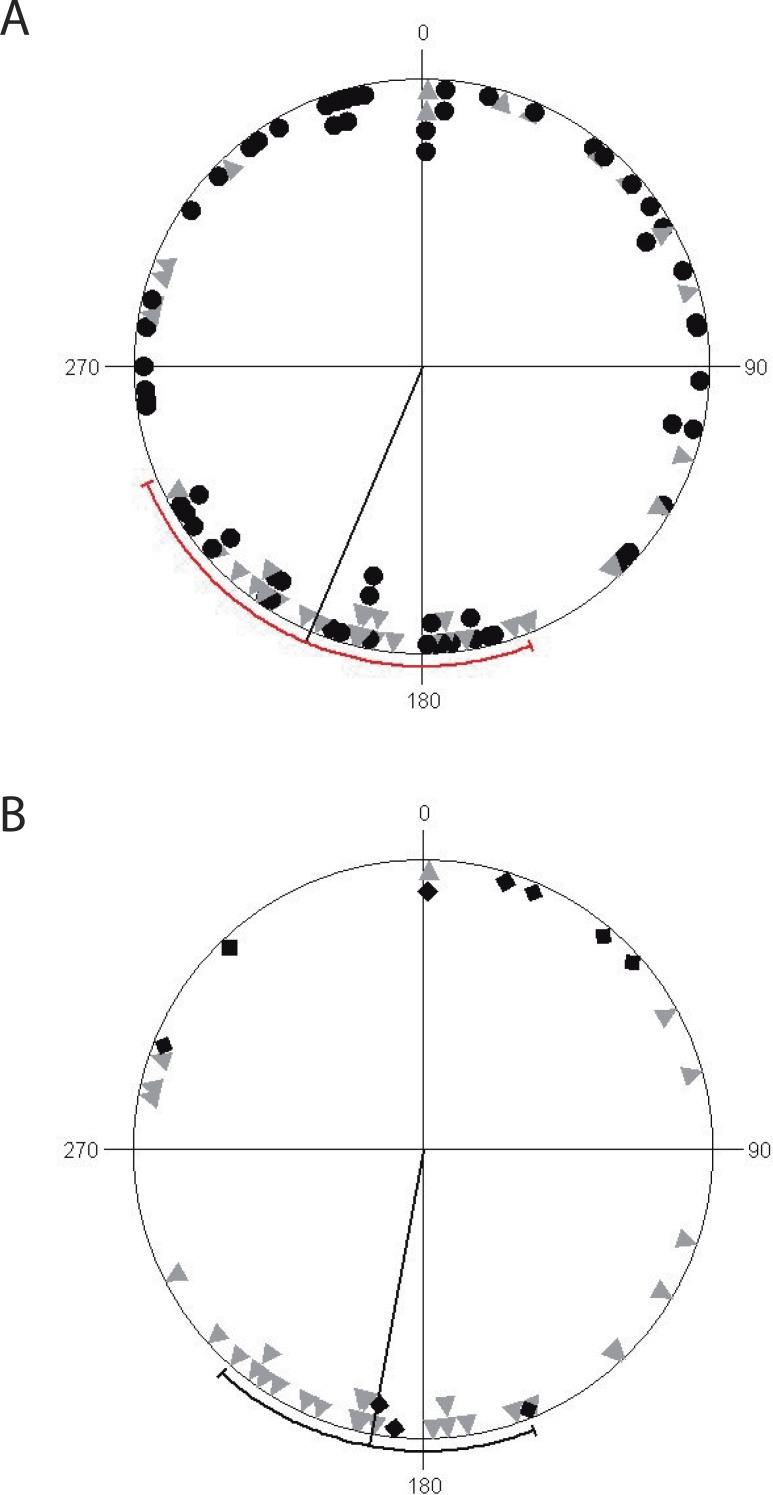
Circular diagrams of ravine orientation along coordinate axes. (A) Distribution of non-reproductive ravines (black circles) and reproductive (gray triangles) with a mean value vector (*μ*) of 246° (±131° SD). (B) Distribution of reproductively active ravines (gray triangles) and inactive (black diamonds) with mean value vector (*μ*) of 190° (±79° SD). The line going from the origin to the outer circumference is the mean vector *μ* for which a 95% confidence interval is plotted as a range along the circle (in red for non-reproductive ravines and reproductive ravines and black for reproductively active and inactive ravines).

Finally, we found that non-reproductive ravines have a uniform orientation along coordinate axes, while reproductive ravines (active and inactive) are non-uniformly orientated but with a significant directionality towards the south, with mean vector μ of 190° ± 79°. (Circular Standard Deviation (CSD)) (*R* = 5.79, *p* = 0.003) (Fig. 3). Reproductively active ravines are not homogeneously distributed around the southern axis (*μ* = 190°± 5° CDS, *R* = 10.54, *p* < 0.0001), while inactive ravines are homogeneously distributed although their orientation is non-significant (*μ* = 12 ± 94° CDS, *R* = 0.65, *p* = 0.53).

### Reproductive success

**Figure 4:**
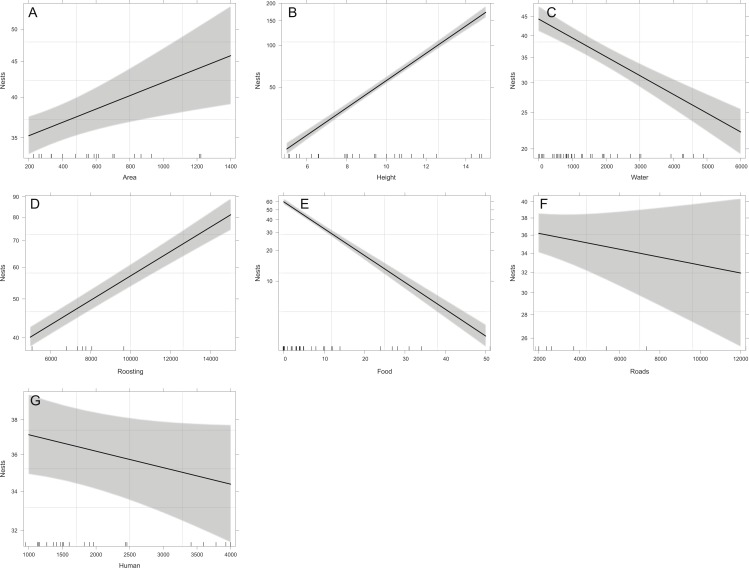
Relationship between the number of nests per ravine and significant environmental variables of reproductive ravines. The values for water, food, roads and human settlements (urbanization) are represented in meters. Lines represent the relationship based on the GLM model that includes all the variables and the shaded area the 95% confidence interval for each case. (A, Area effect plot, B, Height effect plot, C, Water effect plot, D, Roosting effect plot, E, Food effect plot, F, Roads effect plot, G, Human effect plot).

**Figure 5:**
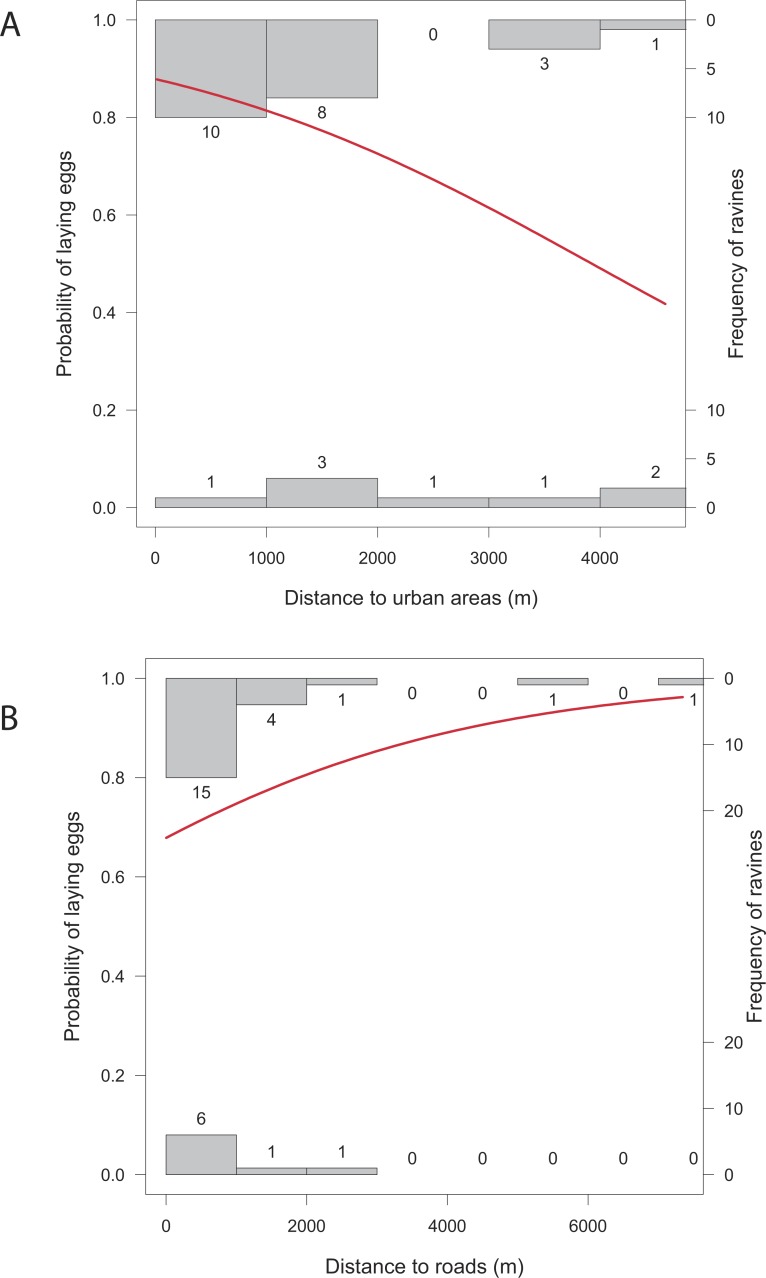
Relationship between the probability of laying eggs vs. significant characteristics of reproductive ravines. (A) Distance to urban areas and (B) distance to roads. The models (in red) are based on GLM models (Logistic regression). Histograms represent the frequency of cases where eggs are present or absent from ravines at different values of the ravine characteristics.

Overall, the number of nests was significantly related to all the studied variables except for distance to roads (*X*^2^ = 1.43, *p* = 0.2318), and distance to human settlements (*X*^2^ = 2.83, *p* = 0.0927). Reproductive ravines with greater height have more nests (*X*^2^ = 858.59, *p* < 0.0001). Those that have greater area (*X*^2^ = 9.44, *p* = 0.0021), that have lower food availability (*X*^2^ = 730.15, *p* < 0.0001), are closer to water bodies (*X*^2^ = 69.37, *p* < 0.0001) and closer to roosting sites (*X*^2^ = 242.68, *p* < 0.0001) also have more nests (Fig. 4). Finally, the probability of producing eggs for an active ravine was significant only with distance to roads (*X*^2^ = 7.04, *p* = 0.0079) and human settlements ( *X*^2^ = 6.80, *p* = 0.0091). Reproductive ravines have a greater probability to produce eggs if they are farther away from roads and closer to human settlements (Fig. 5).

## Discussion

Studies with burrowing birds suggest that ravines tend to be a limited resource due to the particular ravine characteristics required by birds (Heneberg, 2013a). This study is the first to assess the importance of intrinsic and landscape variables for ravine selection as breeding sites. We have also identified ravine characteristics related to greater reproductive success. Patterns found reveal that the Burrowing Parrot mainly selects intrinsic morphological ravine characteristics such as size, orientation and the type of substrate. To a lesser importance individuals tend to choose sites based on extrinsic variables (at the landscape level) such as a location farthest from human disturbance. We found, however, no differences between reproductively active ravines and those that are no longer used for breeding. Selected ravines that have larger size and that are located next to water sources and further away from human disturbances have higher reproductive success.

### Intrinsic morphological characteristics of reproductive ravines

The size of a ravine is a fundamental aspect for reproduction in colonies. Our study shows that from the available pool of ravines, burrowing parrots are selecting those that are larger and taller, which allow individuals to build their nests farther from the ground, probably as a response to terrestrial predation (Corrales et al., 2013). Our results also confirm that the largest ravines are the ones with the highest number of nests and egg production. These findings are consistent with a positive correlation previously found between ravine size and the number of breeding pairs (Tella et al., 2014). One of the reasons why the size of the wall plays a key role in the selection of reproductive ravines is that bigger ravines can host larger breeding colonies, increasing the benefits of group living by way of a dilution effect that reduces predation risk, increases detectability, and enhances deterrence more than smaller ravines Alcock, 2012. The adaptive advantage of group living has been demonstrated for gregarious species where a larger colony size increases the benefits it offers in terms of foraging efficiency and reduced predation risk (Lack, 1968; Wittenberger & Hunt, 1985; Sachs et al., 2007). In addition, there may be an association between a colonial nesting strategy, scarce in other Psittacidae, and reproductive ontogeny because lower predation rates allow greater brood development time compared to other similar parrots (Masello & Quillfeldt , 2002). Our study is the first to show that larger colonies have a higher reproductive success as observed in two consecutive seasons. Future studies should address if a higher reproductive success is also associated to the advantages of group life, and whether predation or foraging strategies changes across colonies of different sizes.

Physical soil characteristics of reproductive ravines are also crucial for burrowing birds (Heneberg & Simecek, 2004). The presence and distribution of certain substrates has been suggested as a driver of the presence of European burrowing birds because of the correlation between the distribution patterns of sediments and nesting areas (Wesolowski, 2007). Moreover, nest construction leads to high-energy expenditure that is conditional to the type of substrate in which construction takes place. Thus, particle size is essential given the “Heneberg compromise” which establishes a trade-off between tunnel stability (reasonable strength of substrate) and the ease of drilling (cohesion) and construction (Martin, Aitken & Wiebe, 2004; Smalley et al., 2013a). In this sense, our study shows that the type of substrate is a determining factor in the selection of reproductive ravines and the substrate more preferable is sand with stones of small size present in ninety percent of reproductive ravines. This type of substrate is predominant in ravines whose morphodynamic origin is colluvial deposits; however, most reproductive ravines with this material are present in cones of dejection (Fig. S1). This means that despite having a greater availability of ravines with a suitable substrate in the form of colluvial deposits, individuals prefer this type of substrate in cones of dejection. The combination of these features may provide a series of benefits not provided by deposits with different origin. Alluvial terraces are uncommon in the study area, there are only three formations and all are reproductively active and present a very fine sedimentary substrate composed of sand and clay in lesser proportion, which originates from sporadic heavy rainfall scenarios, slightly recurrent in this type of ecosystem (Cepeda, 2009).

Studies with burrowing bird species have determined a correlation between soil particle size and structure of the nest tunnel, where longest tunnels are formed by substrates with smaller particles positively affecting breeding success (Heneberg, 2009; Yuan et al., 2006). Substrates with low particle size confer tunnel stability, ease of excavation due to their metastable nature and allow gas movement. In addition, these optimal substrates must have low clay content (less than 10%) for good compaction (neither too strong nor too soft) in order to be resistant to penetration (Heneberg, 2009; Heneberg, 2013a; Smalley et al., 2013a; Smalley et al., 2013b). Studies at the specific substrate level are needed in Neotropical burrowing birds because nesting substrates have a different origin then those used by European burrowing birds, and could have other relevant physical characteristics not previously evaluated.

In cavity-nesters, nest selection is guided in part by the correlation between the resultant microclimate inside the nest and its orientation because the thermal stability provided by substrate characteristics and cavity orientation influences their reproductive success (Hooge, Stanback & Koenig, 1999; Wiebe, 2001; Ardia, Perez & Clotfelter, 2006; White, Brown & Collazo, 2006; Salaberria et al., 2014). Due to the arid environment in which *C. patagonus bloxami* is distributed, the choice of the ravine and the place of the cavity must be precise in order to maintain a stable microenvironment for proper brood development. Because this species is located in the southern hemisphere, north facing slopes (equatorial exposure), exhibit greater insolation than south facing slopes (polar exposure) that are usually less dry (Cepeda, 2009). In fact, the majority of reproductive ravines were located facing south (polar exposure), were shade is prevalent all day and where the temperature of the wall remains more stable compared to north facing walls (equatorial exposure). Therefore, ravines with equatorial exposure tend to be susceptible to reaching high temperatures because of the intense radiation that in turn makes them unviable for nesting. This explanation is in accord with the chosen directionality found in reproductive ravines (i.e., slopes of polar exposure). Furthermore, this directionality is most prevalent in reproductively active ravines. Choosing ravines with equatorial exposure usually ends in reproductive failure. For example, it is common for young inexperienced pairs to construct nests in ravines with non-optimal orientation. These ravines do not provide a stable microclimate and are unviable for brood development resulting in nest desertion. We have been able to observe such events in the field across seasons. After failed events, ravines are not used by the same breeding pair the following season. These nests represent a portion of those nests that are found on ravines that are not facing directly south. Similar results have been found in studies of forest excavators, where individuals nesting in warmer holes were more likely to be successful (Wiebe, 2001; Ardia, Perez & Clotfelter, 2006). An additional strategy in order to avoid high temperatures in this type of extreme dry ecosystem could be depth of the incubation chamber present in the cavity. Although deeper chambers would have lower incidence of radiation, there are other parameters that need to be taken into account, such as structural stability or gas exchange with outside (Wiebe & Swift, 2001). The burrowing parrot builds its nest forming one to three meter galleries that end in an incubation chamber without any substrate (Vargas-Rodríguez & Squeo, 2014). Depth variability may be associated to the external temperature of the ravine or to its degree of isolation. Further evaluation of the influence of nest orientation and gallery length on the thermal variation within the incubation chamber and its influence on fitness is merited.

### Extrinsic ravine characteristics (landscape level)

Our results show that the presence of anthropogenic elements within the landscape significantly influences the selection of reproductive ravines at a large scale. For example, ravines with the highest number of nests tend to be closer to roads and human settlements, although only ravines with greater reproductive success (i.e., more eggs production) are closer to human settlements. This inconsistency in the pattern can be explained by the topographical relief of the area, which makes proximity to human elements unavoidable. Reproductive ravines have a fluvial origin and therefore, tend to be located within small canyons that also run along orographic elements where roads that lead into the Andean foothills have been constructed. Therefore, proximity to roads and human settlements is unavoidable in most cases. The overlap of human influence and reproductive ravines could have important consequences for the viability and continuity of reproductive colonies. There are numerous studies that address the effects of human perturbation on nesting success in this species (e.g., Masello & Quillfeldt, 2002; Masello & Quillfeldt, 2004). They report that human disturbance negatively influences nest success, especially during the incubation period and the first week after hatching, with effects that can lead to abandonment of the nest. Other studies, however, show that there is a high inter-individual variability in the fear against humans and thus, a large potential for dealing well with an anthropic disturbance event as described for other species (Carrete & Tella, 2011). In fact, in this study we report that *C. patagonus bloxami* has the ability to use artificial ravines for reproduction; moreover, all artificial reproductive ravines produced eggs. Therefore, newly created ravines are being exploited by the colonies, and these ravines may contain malleable and suitable substrates for nesting. This has also been reported in Argentina where large nesting colonies are located in urban centers that provide artificial ravines (pits or quarries) (Masello et al., 2011; Tella et al., 2014). This type of behavior has been referred to as an innovation for the species.

The fact that food is not a significant variable indicates that the presence of items near ravines is not relevant for choosing them as sites for reproduction. Disposition of the colony in space is not related to the proximity to the food, which was also observed in other digging birds, where there was no relation between colony size and food availability (Hoi, Kristofik & Darolov, 2015). This pattern is in accord with previous studies on this species in Argentina and Chile, where foraging groups are able to move between 40 km and 60 km one to four times a day from the ravine to foraging areas (Masello & Quillfeldt, 2012; Vargas-Rodríguez & Squeo, 2014). Nevertheless the proximity of water to ravines is key for site-selection. Water bodies are places that not only provide water, but that also act as meeting places for the different colonies. The arid conditions around the habitat makes the availability of natural watersheds scarce or absent in some areas. Therefore, colonies in the area depend entirely on water that is available near human settlements. This foraging behavior is common in the area, where individuals use water sources daily (Vargas-Rodríguez & Squeo, 2014). This could in part explain why ravines with more egg production are closer to human settlements.

Because initially parrots selected ravines for reproduction following the criteria identified in this study, active and inactive ravines should have similar characteristics explaining the lack of significant differences between reproductively active and inactive ravines. Their subsequent inactivity may be due to factors not evaluated in our study, such as predation, competition or by the population decline that has suffered. Subsequent ravine inactivity may be an exploratory phenomenon of juvenile pairs as described above. We have observed the formation of a new active ravine composed of a single pair on a wall of small size, polar exposure and located at the edge of a busy road. The couple incubated two eggs but broods did not survive. The following year, the couple did not return to the nest. Similar behavior has also been reported by Tella et al. (2014) and reported for facultative colonial birds, where solitary pairs can colonize new nesting sites and establishes a new colony.

## Conclusions

Nest site-selection is a complex activity for cavity nesting birds. We show that both intrinsic characteristics of ravine and landscape elements determine the choice of nesting sites. The difference in selection between intrinsic and extrinsic variables seems to be conditioned by the orography of the nesting habitat, which determines the availability and disposition of resources and disturbances in space. The area is a system of gullies of fluvial origin where ravines are located along them, in addition to available water bodies, human settlements and roads. Other resources such as food and roosting, however, are located outside the old riverbed. In this landscape of diverse ravines and dispersed resources, known as partial habitats (*sensu* Westrich, 1996), it seems that individuals first choose areas based on landscape level variables, selecting specific areas where the necessary resources for nesting are concentrated (i.e., gullies where there are ravines, water bodies, roosting and food concentrated). Subsequently, ravines are selected based on the characteristics described in this study; size, substrate, geological origin and orientation. Finally, a decision to reuse the ravine is made based on reproductive success. If the chosen ravine favors reproduction, it will continue to be used year after year, increasing the size of the colony and the number of nests and egg production. The different selection scales observed are in accord with behavioral patterns predicted by foraging theory, which proposes that individuals perform different choices depending on the scale. At the landscape level individuals should choose the optimal gully and subsequently, within gullies optimal ravines should be chosen for reproduction based on their characteristics.

These aspects are fundamental for conservation strategies that are currently being carried out for this species, which is focused of the conservation and/or construction of artificial ravines for nesting (Vargas-Rodríguez & Squeo, 2014). They do not take into account, however, aspects associated to the availability and proximity to resources and disturbances, which as demonstrated in this study, are fundamental elements for fitness. Future studies should incorporate not only ravine characteristics fundamental for the nesting of the species, but also the elements at the landscape level to effectively conduct conservation actions for this threatened subspecies.

##  Supplemental Information

10.7717/peerj.3182/supp-1 Figure S1 Proportion of all study ravines Proportion of all study ravines (in black non-reproductive ravines and gray reproductive) (A, B) and reproductive ravines (in black inactive reproductive ravines and gray active) (C, D) in relation to their corresponding geomorphology characteristics to substrate (A, C) and geological origin (B, D) expressed in percentages in each study group. The absolute values of each category of study, type of substrate and origin. (APG, sand with big stones; APM, sand with medium stones; APP, sand with small stones; A, sand without stones; CD, cones of dejection, DC, colluvial deposits, FL, flow sandy slope, TA, alluvial terrace; TE, talud erosion).

10.7717/peerj.3182/supp-2 Supplemental Information 2 Mean values, standard deviation, confidence intervals (italics) and effect size *θ* of the ravine characteristics Mean values, standard deviation, confidence intervals (italics) and effect size *θ* of the ravine characteristics (effect size comparisons are for Non-reproductive ravines vs. Reproductive ravines and Active ravines vs. Inactive ravines; given by *θ* = (*μ*_1_ − *μ*_2_)∕*σ*_1_). The nature of the ravine (origin geological or artificial of the ravine) is expressed for better viewing in proportion to total number of artificial ravines for each category. Numbers in parentheses correspond to the number of ravines in each category that presented this characteristic. (APG, sand with big stones; APM, sand with medium stones; APP, sand with small stones; A, sand without stones; CD, cones of dejection, DC, colluvial deposits, FL, flow sandy slope, TA, alluvial terrace; TE, talud erosion)

10.7717/peerj.3182/supp-3 Data S1 Raw data file of study variables Geological origin (APG, sand with big stones; APM, sand with medium stones; APP, sand with small stones; A, sand without stones; CD, dejection cones, DC, colluvial deposits, FL, flow sandy slope, TA, alluvial terrace; TE, talud erosion).

## Additional Information and Declarations

### Data Availability

The following information was supplied regarding data availability: The raw data has been supplied as a Supplementary File.
