# Supplementary material for: The importance of scale-dependent ravine characteristics on breeding-site selection by the Burrowing Parrot, Cyanoliseus patagonus
Source: PeerJ. 2017 Apr 26;5:e3182. doi: 10.7717/peerj.3182 (PMC5408729; doi:10.7717/peerj.3182)
Supplement: Figure S1 — Proportion of all study ravines (in black non-reproductive ravines and gray reproductive) (A, B) and reproductive ravines (in black inactive reproductive ravines and gray active) (C, D) in relation to their corresponding geomorphology characteristics to substrate (A, C) and geological origin (B, D) expressed in percentages in each study group. The absolute values of each category of study, type of substrate and origin. (APG, sand with big stones; APM, sand with medium stones; APP, sand with small stones; A, sand without stones; CD, cones of dejection, DC, colluvial deposits, FL, flow sandy slope, TA, alluvial terrace; TE, talud erosion). [file peerj-05-3182-s001.pdf]

Supplemental Information

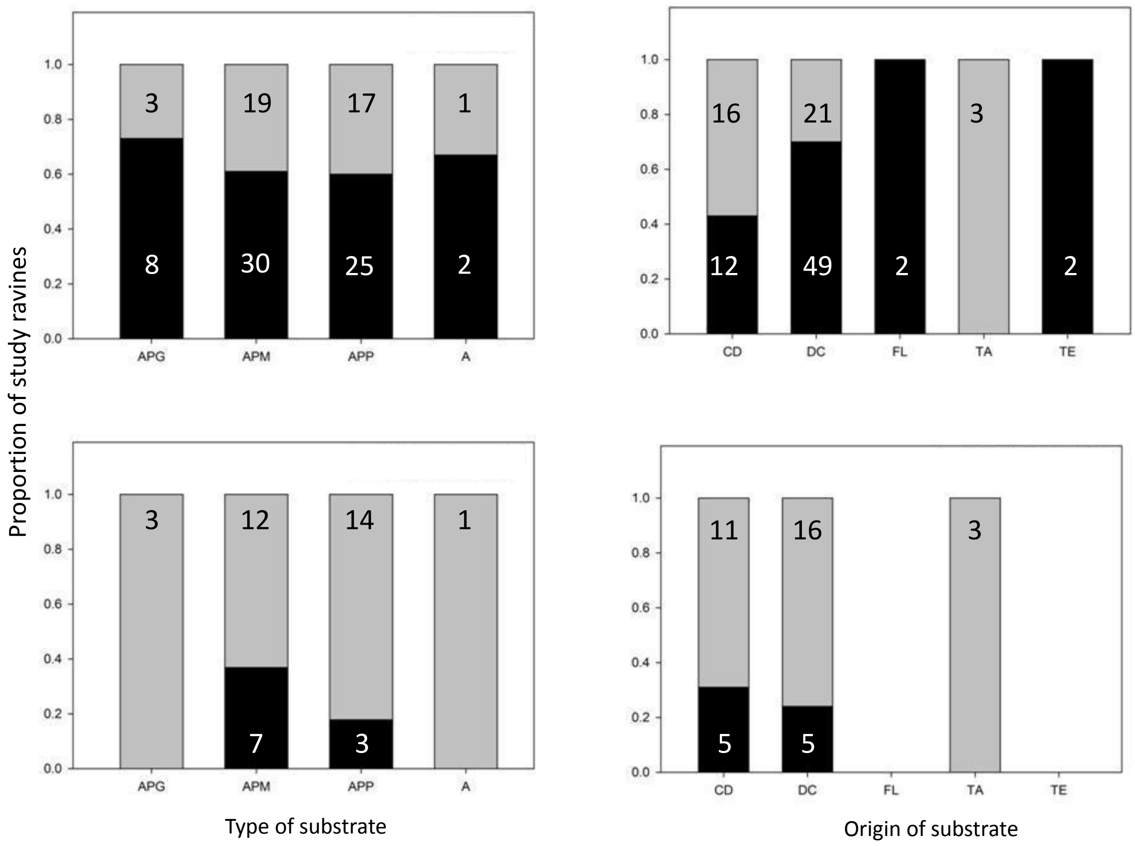

Figure S1. Proportion of all study ravines (in black non-reproductive ravines and gray reproductive) (A, B) and reproductive ravines (in black inactive reproductive ravines and gray active) (C, D) in relation to their corresponding geomorphology characteristics to substrate (A, C) and geological origin (B, D) expressed in percentages in each study group. The absolute values of each category of study, type of substrate and origin. (APG = sand with big stones; APM = sand with medium stones; APP = sand with small stones; A = sand without stones; CD = cones of dejection, DC = colluvial deposits, FL = flow sandy slope, TA = alluvial terrace; TE = talud erosion).
