# Supplementary material for: The importance of scale-dependent ravine characteristics on breeding-site selection by the Burrowing Parrot, Cyanoliseus patagonus
Source: PeerJ. 2017 Apr 26;5:e3182. doi: 10.7717/peerj.3182 (PMC5408729; doi:10.7717/peerj.3182)
Supplement: Supplemental Information 2 — Mean values, standard deviation, confidence intervals (italics) and effect size θ of the ravine characteristics (effect size comparisons are for Non-reproductive ravines vs. Reproductive ravines and Active ravines vs. Inactive ravines; given by θ = (μ1 − μ2)∕σ1). The nature of the ravine (origin geological or artificial of the ravine) is expressed for better viewing in proportion to total number of artificial ravines for each category. Numbers in parentheses correspond to the number of ravines in each category that presented this characteristic. (APG, sand with big stones; APM, sand with medium stones; APP, sand with small stones; A, sand without stones; CD, cones of dejection, DC, colluvial deposits, FL, flow sandy slope, TA, alluvial terrace; TE, talud erosion) [file peerj-05-3182-s002.docx]

**Supplemental Information**

**Table S1**. Mean values, standard deviation, confidence intervals (italics) and effect size θ of the ravine characteristics (Effect size comparisons are for Non-reproductive ravines vs. Reproductive ravines and Active ravines vs. Inactive ravines; given by θ = (µ_1_-µ_2_)/σ_1_). The nature of the ravine (origin geological or artificial of the ravine) is expressed for better viewing in proportion to total number of artificial ravines for each category. Numbers in parentheses correspond to the number of ravines in each category that presented this characteristic. (APG = sand with big stones; APM = sand with medium stones; APP = sand with small stones; A = sand without stones; CD = cones of dejection, DC = colluvial deposits, FL = flow sandy slope, TA = alluvial terrace; TE = talud erosion).

| **Variable** | **Non-reproductive ravines** | **Reproductive ravines** | **Effect size θ** | **Active reproductive ravines** | **Inactive reproductive ravines** | **Effect size θ** |
| --- | --- | --- | --- | --- | --- | --- |
| **Slope (pitch)** | 73.1 (± 12.57)(*±3.01*) | 82.87 (± 7.14)(*± 2.21*) | -1.37 | 82.95 (± 6.34)(*±2.65*) | 82.06 (± 9.41)(*±4.36*) | 0.14 |
| **Area (m^2^)** | 223.54 (± 241.31)(*±59.12*) | 368.45 (± 382.9)(*± 118.66*) | -0.38 | 387.57 (±349.06)(*±145.86*) | 311.1 (± 487.67)(±225.29) | 0.22 |
| **Height (m)** | 6.8 (± 3.95)(*±0.97*) | 8.13 (± 4.2)(*± 1.3*) | -0.32 | 8.91 (± 4.13)(*±1.73*) | 5. 81 (± 3.73)(*±1.72*) | 0.75 |
| **Water distance (m)** | 2406. 36 (± 1773.4)(*±434.48*) | 166.363 (± 1590)(*± 492.75*) | 1.41 | 1645.89 (± 1680)(*±702.02*) | 1715.8 (± 1362.6)(*±629.49*) | -0.04 |
| **Roosting distance (m)** | 3003.23 (± 1988.33)(*±487.14*) | 3682.8 (± 4470,2)(*± 1385.33*) | -0.15 | 4360.78 (± 4949)(*±2068.055*) | 1648.86 (± 1241,9)(*±573.73*) | 0.55 |
| **Road distance (m)** | 565. 52 (± 1199.37)(*±293.85*) | 1190.34 (± 2367.83)(*± 733.8*) | -0.26 | 1342.86 (± 2627.28)(*±1097.87*) | 732.81 (± 1320.45)(*±610.02*) | 0.23 |
| **Urban distance (m)** | 1820.83 (± 1391. 39)(*±340.89*) | 1595.27 (± 1369.2)(*± 424.32*) | 0.16 | 1713.45 (± 1438. 11)(*±600.95*) | 1240.73 (± 1128)(*±521.11*) | 0.32 |
| **Food abundance (m)** | 9. 05 (± 13,28)(*±3.25*) | 8.15 (± 11.5)(*± 3.6*) | 0.08 | 8.63 (± 11. 82)(*±4.94*) | 6. 7 (± 11. 15)(*±5.15*) | 0.16 |
| **Substratum (m)** | APM (30) A(2)APG (8)APP(25) | APM(19) A(1) APG (3) APP(17) | - | APG (3) APM(12) APP(14)A(1) | APG(0) APM(7) APP(3) | - |
| **Geological origin** | CD(12) DC(49) FL(2) TA (0) TE (2) | CD(16) DC(21) FL (0) TA(3) TE(0) | - | CD(11) DC (16) TA (3) | CD(5) DC(5) | - |
| **Nature of the ravine.** | 8/40 | 9/65 | - | 8/30 | 0/10 | - |
