# Supplementary material for: The importance of scale-dependent ravine characteristics on breeding-site selection by the Burrowing Parrot, Cyanoliseus patagonus
Source: PeerJ. 2017 Apr 26;5:e3182. doi: 10.7717/peerj.3182 (PMC5408729; doi:10.7717/peerj.3182)
Supplement: Data S1 — Geological origin (APG, sand with big stones; APM, sand with medium stones; APP, sand with small stones; A, sand without stones; CD, dejection cones, DC, colluvial deposits, FL, flow sandy slope, TA, alluvial terrace; TE, talud erosion). [file peerj-05-3182-s003.pdf]

ID	Category	Orientation	Slope	Geological origin	Substratum	Area
1	Reproductive Ravine	NNE	77.1	DC	APM	98.65
2	Reproductive Ravine	S	88.4	CD	APP	261.24
3	Reproductive Ravine	SSO	87.7	CD	APM	1222.98
4	Reproductive Ravine	SSE	80.3	CD	APP	276.93
5	Reproductive Ravine	S	82.5	DC	APM	827.53
6	Reproductive Ravine	N	84.5	CD	APM	867.25
7	Reproductive Ravine	SE	88.2	DC	APM	707.68
8	Reproductive Ravine	S	77.2	DC	APM	929.35
9	Reproductive Ravine	SSO	64.5	CD	APP	103.77
10	Reproductive Ravine	S	87.1	CD	APP	83.11
11	Reproductive Ravine	ESE	81.7	CD	APP	588.07
12	Reproductive Ravine	SSO	88.1	DC	APP	334.29
13	Reproductive Ravine	SSE	73.6	CD	APP	399.84
14	Reproductive Ravine	ESE	88.5	DC	APP	126.76
15	Reproductive Ravine	SSE	87.3	DC	APG	333.08
16	Reproductive Ravine	S	78.5	DC	APM	601.57
17	Reproductive Ravine	SE	95.7	DC	APG	99.64
18	Reproductive Ravine	NO	87.9	DC	APM	80.71
19	Reproductive Ravine	ONO	57.7	CD	APM	50.77
20	Reproductive Ravine	NE	85.1	DC	APP	35.2
21	Reproductive Ravine	SO	84.7	DC	APP	147.59
22	Reproductive Ravine	ONO	87.7	CD	APP	108.95
23	Reproductive Ravine	NE	89.1	DC	APM	25.14
24	Reproductive Ravine	SO	87.8	CD	APM	228.36
25	Reproductive Ravine	ENE	82.8	TA	A	119.53
26	Reproductive Ravine	SSO	80.6	DC	APM	97.39
27	Reproductive Ravine	ONO	87.1	CD	APM	1213.03
28	Reproductive Ravine	S	82.6	CD	APM	95.69
29	Reproductive Ravine	NNE	89.2	DC	APM	1497.54
30	Reproductive Ravine	SO	87.7	TA	APP	554.38
31	Reproductive Ravine	SSO	86.5	CD	APP	613.99
32	Reproductive Ravine	SO	81.1	CD	APM	72.86
33	Reproductive Ravine	OSO	79.9	DC	APM	74.69
34	Reproductive Ravine	ENE	88.1	DC	APM	545.45
35	Reproductive Ravine	SSO	85.1	TA	APM	74.65
36	Reproductive Ravine	O	83.1	CD	APP	700.17
37	Reproductive Ravine	S	79.9	DC	APP	477.03
38	Reproductive Ravine	N	77.1	DC	APP	45.6
39	Reproductive Ravine	SO	73.1	DC	APP	6.97
40	Reproductive Ravine	SSO	74.3	DC	APG	10.56
41	No reporductive Ravine	ENE	88.7	DC	APP	139.26
42	No reporductive Ravine	SO	83.1	DC	APP	845.88
43	No reporductive Ravine	S	54.1	DC	APG	98.12
44	No reporductive Ravine	NNO	72.22	DC	APM	63.18
45	No reporductive Ravine	S	77.5	DC	APP	60.18
46	No reporductive Ravine	S	73.1	DC	APM	22.48
47	No reporductive Ravine	N	63.7	DC	APP	95.82
48	No reporductive Ravine	NNO	74.7	DC	APP	709.50
49	No reporductive Ravine	SSO	81.2	DC	APG	122.43

50	No reporductive Ravine	N	61.3	TE	APG	227.28
51	No reporductive Ravine	ENE	64.7	DC	APM	225.11
52	No reporductive Ravine	SE	64.4	DC	APM	484.07
53	No reporductive Ravine	N	88.1	DC	APM	427.01
54	No reporductive Ravine	NNE	73.2	DC	APM	68.60
55	No reporductive Ravine	SSO	77.1	CD	APM	67.89
56	No reporductive Ravine	NE	74.3	CD	APG	699.74
57	No reporductive Ravine	SSO	54.4	CD	APP	258.24
58	No reporductive Ravine	S	84.1	CD	APP	515.08
59	No reporductive Ravine	NO	84.5	CD	APG	341.34
60	No reporductive Ravine	SE	60.3	DC	APP	147.81
61	No reporductive Ravine	SO	73.5	DC	APM	133.31
62	No reporductive Ravine	ENE	68.8	CD	APP	52.75
63	No reporductive Ravine	NO	86.1	DC	APM	83.06
64	No reporductive Ravine	NNO	52.7	CD	APP	8.17
65	No reporductive Ravine	NE	71.1	DC	APM	192.46
66	No reporductive Ravine	NO	86.8	CD	APM	33.60
67	No reporductive Ravine	SO	61.1	TE	APP	629.50
68	No reporductive Ravine	NNO	66.5	DC	APP	643.78
69	No reporductive Ravine	SSO	7.52	CD	APM	159.96
70	No reporductive Ravine	O	61.1	DC	APP	397.36
71	No reporductive Ravine	NO	80.4	DC	APP	40.33
72	No reporductive Ravine	NNO	65.9	DC	APG	203.70
73	No reporductive Ravine	O	86.3	DC	APM	14.07
74	No reporductive Ravine	NNE	75.7	DC	APM	108.15
75	No reporductive Ravine	S	73.5	DC	APM	938.04
76	No reporductive Ravine	SSO	81	CD	A	138.65
77	No reporductive Ravine	SSO	64.8	DC	APM	491.41
78	No reporductive Ravine	SSE	60.1	DC	APP	982.80
79	No reporductive Ravine	S	65.5	DC	APM	504.30
80	No reporductive Ravine	NE	71.1	DC	APP	13.68
81	No reporductive Ravine	OSO	77.3	DC	APP	74.11
82	No reporductive Ravine	E	85.3	DC	APP	61.26
83	No reporductive Ravine	ESE	68.2	DC	APM	89.15
84	No reporductive Ravine	OSO	81.3	FL	APP	28.69
85	No reporductive Ravine	S	79.1	DC	APP	17.15
86	No reporductive Ravine	OSO	70.4	FL	APP	10.89
87	No reporductive Ravine	NNO	61.7	DC	APP	35.90
88	No reporductive Ravine	NNO	88.7	DC	APM	88.10
89	No reporductive Ravine	SSE	74	DC	APM	51.34
90	No reporductive Ravine	ONO	84.7	DC	APG	17.63
91	No reporductive Ravine	NNO	77.3	CD	APP	17.39
92	No reporductive Ravine	NE	73.1	DC	APP	78.60
93	No reporductive Ravine	SO	65.1	DC	A	31.20
94	No reporductive Ravine	ESE	88.7	DC	APM	225.97
95	No reporductive Ravine	N	81.2	DC	APG	258.24
96	No reporductive Ravine	NNO	81.1	DC	APM	113.79
97	No reporductive Ravine	E	63.8	CD	APP	213.12
98	No reporductive Ravine	SO	77.1	DC	APM	44.26
99	No reporductive Ravine	E	77.4	DC	APM	426.94

100	No reporductive Ravine	ESE	73.7	DC	APM	102.94
101	No reporductive Ravine	O	71.7	DC	APM	322.54
102	No reporductive Ravine	O	77.7	DC	APM	186.49
103	No reporductive Ravine	SE	77.1	DC	APM	322.00
104	No reporductive Ravine	O	88.1	DC	APM	277.00
105	No reporductive Ravine	S	87.6	DC	APM	47.41

Hight	nest	eggs	Dist.water bodies	Dist. Roosting	Foods	Dist. Road
3.9	3	no	4898	544	5	187
12.52	44	si	4593	502	1	278
18.7	788	si	525	491	3	121
7.92	23	no	659	1961	27	92
11.28	13	no	1539	2746	51	98
8.64	12	no	1930	3233	28	177
10.65	28	si	562	2039	14	90
14.27	109	si	874	1765	8	94
6.58	37	si	119	634	4	42
5.1	32	no	1585	2042	5	193
8.3	18	no	1938	2418	12	435
10.78	144	no	2719	3162	3	100
9.4	219	no	2130	6798	5	3
8.06	15	no	1300	7351	7	1517
14.69	42	si	984	7598	10	1836
5.63	28	no	808	7776	10	2003
5.63	21	si	454	8069	12	2371
3.59	2	no	1061	529	0	45
3.82	6	no	813	588	2	71
3.28	6	no	417	198	0	15
5.49	8	si	729	955	0	52
4.25	23	no	836	1071	2	77
2.46	3	no	876	1097	0	175
6.58	41	si	964	1283	2	52
9.48	183	si	652	9669	0	7344
6.60	9	si	818	1878	24	236
16.08	149	si	4272	4691	31	5340
4.56	101	no	1886	2596	0	2639
14.80	114	no	3034	3701	0	3735
10.43	7	si	4294	416	3	188
11.88	62	no	6870	2520	4	271
5.13	54	si	1274	1516	3	234
4.9	13	si	601	1644	4	387
10.01	27	si	152	1950	8	859
6.23	19	si	0	750	0	756
15.60	141	si	14	19504	34	1378
7.98	156	si	2342	19980	4	317
3.9	5	si	3000	3438	0	103
3.39	2	si	84	5088	0	1440
2.90	6	si	3930	3122	0	12265
6.20	0	no	4037	5060	13	12
13.50	0	no	2732	6247	12	72
5.28	0	no	2014	6963	6	815
3.86	0	no	1850	7027	3	957
3.20	0	no	1769	7054	1	1037
5.59	0	no	4124	4889	13	12
6.14	0	no	2150	2605	10	10
10.50	0	no	1470	1919	9	59
6.62	0	no	1514	1909	7	53

7.26	0	no	772	809	11	178
10.12	0	no	718	2377	10	82
10.97	0	no	1182	1786	0	272
10.38	0	no	1528	1870	12	281
5.80	0	no	1718	1661	5	244
4.29	0	no	2504	489	0	222
11.62	0	no	3409	962	13	301
8.63	0	no	3520	836	0	147
9.86	0	no	5123	735	2	257
6.96	0	no	6484	2166	9	256
5.57	0	no	663	891	0	41
6.48	0	no	800	1028	0	59
2.90	0	no	1120	1346	8	103
4.32	0	no	264	472	0	124
1.26	0	no	237	211	0	141
4.71	0	no	260	70	0	15
2.37	0	no	518	304	2	115
12.80	0	no	1096	1049	12	93
17.81	0	no	129	1362	15	100
4.64	0	no	553	1862	1	83
6.41	0	no	1258	2558	33	59
3.70	0	no	1685	4227	24	69
6.10	0	no	1162	3755	32	87
2.10	0	no	384	3004	12	51
3.50	0	no	1041	1086	23	171
13.75	0	no	991	1044	1	9
6.90	0	no	1168	1643	0	168
8.85	0	no	7463	3220	15	221
13.40	0	no	7232	2908	3	373
7.50	0	no	6960	2659	6	274
1.83	0	no	2830	3259	3	10
4.00	0	no	2554	6387	1	243
5.30	0	no	3130	3565	3	23
4.70	0	no	2884	3336	16	33
2.83	0	no	3983	3940	1	7
4.34	0	no	2146	2599	1	204
1.77	0	no	3983	3940	5	7
2.50	0	no	802	1035	1	158
6.20	0	no	620	1602	2	358
3.48	0	no	3969	4889	13	12
3.37	0	no	1406	7270	7	1407
2.05	0	no	338	187	1	121
5.44	0	no	3932	5368	16	23
5.00	0	no	2754	6150	6	0
9.06	0	no	3784	4177	8	34
8.63	0	no	4037	4873	3	169
4.62	0	no	2893	6158	94	34
7.1	0	no	3930	5285	9	132
3.53	0	no	3213	3565	5	61
10.10	0	no	559	2294	6	1561

8.30	0	no	1649	2888	10	2620
7.30	0	no	2553	3380	32	3487
7.30	0	no	3490	3993	6	4426
10.50	0	no	3846	4362	5	4820
8.90	0	no	4114	4538	8	5057
22.16	0	no	3408	4105	3	4129

Dist.human settlements	ıature of ravine
457	no
428	no
528	no
677	no
1517	si
1904	no
953	no
1268	no
185	no
1600	no
1158	no
1414	no
4343	no
4249	no
3929	no
3782	no
3411	no
122	no
333	no
329	no
800	no
915	no
956	no
1130	no
20	no
1144	si
4586	no
2436	no
3593	no
451	si
2459	no
1365	no
1499	no
1825	no
563	si
16	no
1962	si
1469	si
36	si
4000	si
383	no
4014	no
4558	no
4571	no
4565	no
3047	no
1486	no
1743	no
1721	no

194	no
720	no
1267	no
1525	no
1730	no
2209	no
1131	no
184	no
547	no
2031	no
691	no
828	no
1150	no
400	no
171	no
88	no
348	no
459	no
120	no
539	no
1243	si
1329	no
749	no
10	no
1063	no
1014	no
1186	si
1955	no
2331	no
2582	no
1707	no
4085	no
1817	si
1442	si
1408	si
1311	no
1408	si
886	no
1441	no
3047	no
4361	no
229	no
3752	no
1469	no
3934	si
3183	si
4035	no
132	no
1331	si
768	no

1849	no
2803	no
3786	no
4222	no
4115	no
3952	no
